# Chemical Composition and Comprehensive Antimicrobial Activity of an Ethanolic Extract of Propolis from Tunisia

**DOI:** 10.3390/antibiotics12050802

**Published:** 2023-04-23

**Authors:** Nermine Nefzi, Stefania Pagliari, Luca Campone, Wided Megdiche-Ksouri, Filippo Giarratana, Nicola Cicero, Graziella Ziino, Luca Nalbone

**Affiliations:** 1 Laboratory of Aromatic and Medicinal Plants, Biotechnology Center of Borj-Cédria, BP 901, Hammam-Lif 2050, Tunisia; 2 Faculty of Sciences of Tunis, University of Tunis El Manar, Tunis 1068, Tunisia; 3 Department of Biotechnology and Biosciences, University of Milano-Bicocca, Piazza Della Scienza 2, I-20126 Milano, Italy; 4 Department of Veterinary Sciences, University of Messina, Viale Giovanni Palatucci SNC, 98168 Messina, Italy; 5 RICONNEXIA SRLS, Spin-off of the University of Messina, Viale Giovanni Palatucci SNC, 98168 Messina, Italy; 6 Department of BIOMORF-Biomedical and Dental Sciences and Morphofunctional Imaging, University of Messina, Polo Universitario dell’Annunziata, Viale Palatucci snc, 98168 Messina, Italy

**Keywords:** propolis, biopreservative, tolerance, sustainability, antimicrobial activity, antibiofilm activity, salmon, shelf-life, challenge test, *Listeria monocytogenes*

## Abstract

In the present study, the chemical composition and the in vitro antimicrobial and antibiofilm activity of an ethanolic extract of propolis (EEP) from Tunisia against different ATCC and wild bacterial strains were evaluated. In situ antimicrobial activity and sensory influence of different EEP concentrations (0.5% and 1%), also in combination with 1% vinegar, were evaluated in chilled vacuum-packed salmon tartare. Furthermore, a challenge test was performed on salmon tartare experimentally contaminated with *Listeria monocytogenes* and treated with the different EEP formulations. The in vitro antimicrobial and antibiofilm activity was observed only against Gram-positive bacteria, such as *L. monocytogenes* and *S. aureus*, both ATCC and wild. Results of the in situ analyses revealed significant antimicrobial activity against aerobic colonies, lactic acid bacteria, *Enterobacteriaceae* and *Pseudomonas* spp. only when the EEP was used at 1% and in combination with 1% vinegar. The 1% EEP in combination with 1% vinegar was the most effective treatment also against *L. monocytogenes*, although 0.5% and 1% EEP used alone also showed antilisterial effects. After 7 days of storage, the sensory influence on odor, taste and color of salmon tartare was negligible for all EEP formulations. In this background, results obtained confirmed the antimicrobial efficacy of propolis which could be proposed as a suitable biopreservative to ensure safety and improve the quality of food.

## 1. Introduction

Propolis, also known as bee glue, is a resinous, waxy and viscous substance produced from the exudates of flowers and buds processed by specific enzymes present in the salivary secretions of honeybees (*Apis mellifera* L.) [1]. Bees use propolis both as a thermal insulator for the hives and as an antiseptic to protect the larvae, honey stores and combs from pathogenic microorganisms acting as a physical barrier and immune modulator [2,3]. Its use as a dietary supplement has greatly increased during the last years thanks to its numerous beneficial effects on health [4]. Overall, propolis contains about 45–50% plant resins, 25–30% wax, 10% essential oils, 5% pollen and 5% micro- and macronutrients (Ca, Mg, Fe, Z, Na, K, P, Mn, Co) and vitamins (A, B1, B2, B3, C, E, H and P) [5]. However, the chemical composition of propolis can vary greatly, mainly depending on geographical and botanical origin and the harvest season [6,7]. In this regard, more than 420 different chemical constituents have been identified in propolis [8,9]. Most of its biological activities can be attributed to flavonoids (e.g., chrysin, pinocembrin, apigenin, galangin, kaempferol, quercetin, tectochrysin, pinostrobin, and pinobenchin) which are extensively present in propolis, followed by phenolic acids (caffeic, cinnamic and ferulic, benzoic, salicylic and *p*-coumaric acids), aromatic esters (artepillin C, caffeic and cinnamic acid esters) and terpenoids (camphor, terpineol, geraniol, nerol, and farnesol) [10]. This complexity and variability of the chemical composition underlie the numerous biological properties that have been attributed to propolis such as the antioxidant, antibacterial, antiviral, antiparasitic, anti-inflammatory, anti-tumoral, hepatoprotective and antidiabetic activity [11]. Given the antimicrobial properties, the use of propolis was proposed as a natural preservative in foods against spoilage microorganisms to extend their shelf-life or against different foodborne pathogens [12]. This is in line with new consumer trends that, over the last few years, have increasingly demanded natural alternatives to conventional synthetic additives used in food production [13,14]. Propolis has been used in foods according to different formulations and the use of ethanolic extracts is one of the most proposed [15]. The use of ethanolic extracts, however, finds some constraints mainly related to the influence on the sensory characteristics of the products which may not be appreciated by consumers. Furthermore, the addition of an alcoholic component to foods could discourage their consumption for health and religious reasons. To overcome these limitations, the use of polymeric microencapsulates of propolis applied directly on the food or as a packaging coating has been proposed [16]. However, if on one hand the microencapsulation technique significantly reduces the sensory influence on food, on the other hand, it may not be easy to apply at an industrial level due to production costs and processing times. As reported in a recent and comprehensive review conducted by Pobiega et al. [10], although several promising studies are currently available in the literature on the use of propolis as a natural preservative in food, further research is still needed to understand how it can be used on an industrial scale. In the present study, the chemical composition and antimicrobial activity of an ethanolic extract of Tunisian propolis were evaluated. The antimicrobial efficacy was assessed both in vitro and directly in food, evaluating its use to extend the shelf life of salmon tartare and in a challenge test against *Listeria monocytogenes*.

## 2. Results

### 2.1. Chemical Composition of Ethanolic Extract of Propolis

UHPLC-ESI/HRMS analysis was used in order to characterize the main bioactive compounds of EEP. Identification and assignment of individual phenolic compounds were determined according to the corresponding MS spectra [M–H]^−^, accurate mass, characteristic fragmentation, and to the literature findings. The LC-MS/MS analysis revealed the presence of 28 phenolic compounds in the EEP, including phenolic acids, flavones, flavanones, flavonols and their derivatives as well as terpenoids. Detailed information about the compounds was summarized in Table S1.

#### 2.1.1. Phenolic Acids

Four *p*-coumaric acid derivatives (**3**, **18**, **19** and **25**) were tentatively identified in the present study. According to the precursor ions [M–H]^−^ at *m*/*z* 359.19 and 383.39, compounds **3** and **25** pointed to the molecular formula C_19_H_20_O_7_ and C_21_H_20_O_7_, respectively. Compounds **18** and **19** matched exactly with the accurate mass at *m*/*z* 325.19 and the same fragment ions *m*/*z* 187, *m*/*z* 163 and *m*/*z* 145. Of the compounds with incomplete identification, these *p*-coumaric acid derivatives were identified by the specific fragments of 163 [C_9_H_7_O_3_–H]^-^, 145 [C_9_H_5_O_2_–H]^-^ and 119 [C_9_H_7_O_3_–H–CO_2_]^-^ [17]. Caffeoyl quinic acid (4) was identified by comparing our findings with those of a previous report [18] yielding product ions at *m*/*z* 179 and *m*/*z* 191 corresponding to the losses of caffeic acid and quinic acid fragment. Compound **20** (Rt = 12.43 min.) with the precursor ion at *m*/*z* 385.20 was identified as a Ferulic acid dimer. MS^2^ spectral data showed non-specific product ions at *m*/*z* 341, 282 and 193. The *m*/*z* 341 and 282 can be related to the loss of CO_2_, CH_3_ and CH_2_O, as reported by Silva et al. [19]. The characteristic product ion at *m*/*z* 193 corresponds to the cleavage of the ether link between the two monomeric units and the elimination of a neutral ferulic acid. Compound **21** (Rt = 12.95 min.) generated a peak base [M–H]^−^ ion at *m*/*z* 347.22 and was tentatively identified as a gallic acid derivative by comparing our findings with those of a previous report [20], where this compound yielding product ions at *m*/*z* 169, 125 and 124. The corresponding 169 fragments correspond to the gallic acid moisture [20].

#### 2.1.2. Flavones

Among the flavonoids detected in the propolis extract, four were flavones. Compound **6** (Dihydroxy-dimethoxyflavone) was detected with [M–H]^−^ at *m*/*z* 315.0875. It was identity-based on the fragment ions at *m*/*z* 255 [M–H–60] and *m*/*z* 151 [M–H–164] and confirmed by comparing them with data from a previous study [21]. Compound **8** was identified as Hispidulin (Rt = 8.96 min.) and exhibited a peak [M–H]^−^ ion at *m*/*z* 299 with diagnostic fragments at *m*/*z* 255 due to [M–H–CO_2_] and 227 [M–CO_2_–CO], respectively [21]. Compound **9** was tentatively identified as Tricin. The identification of this substance was based on their chromatographic behavior and production at *m*/*z* 299 attributed to [M–H–2CH_3_], 271 attributed to [M–H–C_2_H_2_O_2_] and 243 attributed to [M–H–C_4_H_6_O_2_]. Compound **12** (Rt = 10.41 min.) was identified as Chrysin, showing an ion at *m*/*z* 253.0495 and a fragment ion at *m*/*z* 119 [M–H–139] [21].

#### 2.1.3. Flavanones

Dimethoxy-naringenin diglucoside (1) was suggested for the precursor ion at *m*/*z* 623.20 which in turn gave fragments at *m*/*z* 271 through the loss of two methyl groups from aglycon part [18]. Compound **2** occurred at Rt of 6.71 min. and exhibited a [M–H]^−^ ion at *m*/*z* 609.1854. The full MS spectra of this peak allowed the molecular formula C_28_H_34_O_15_ to be tentatively identified as hesperidin [22]. Compound **13** displayed the [M-H]^-^ ions at *m*/*z* 255.0662. MS/MS spectral data show the product ions at *m*/*z* 213 and 151. Compound **13** was proposed as pinocembrin [23].

#### 2.1.4. Flavonols

Compound **7** was identified as pinobanksin (Rt = 8.67 min) with precursor ions at *m*/*z* 271.0610 accompanied by two intense product ions at *m*/*z* 253 accounting for the [M–H–H_2_O]^-^ and 151 [24]. Compounds **5**, **14**, **16** and **17** displayed behaviors similar to those of compound **7**. They all produced ions corresponding to the MS/MS spectrum of pinobanksin and were identified as pinobanksin derivatives compounds. As described in several reports, the esterification of this compound occurs preferably at C-3. Compounds **5**, **14**, **16** and **17** were assigned as pinobanksin-methyl-ether, pinobanksin-acetate, pinobanksin-propionate and pinobanksin-3-O-pentanoate, respectively. All pinobanksin esters shared a fragmentation pathway that was consistent with the literature data [18,25]. By MS/MS analysis, all generated abundant ions at *m*/*z* 271 accounting for [M–acyl group] and the ion at *m*/*z* 253 accounting for [M–acyl group–H_2_O]^-^. Compounds **10** and **11** eluting at 9.34 and 10.35 respectively displayed the same [M–H]^−^ ions at *m*/*z* 335.26 and pointed to the molecular formula C_15_H_12_O_9_. The fragment ion *m*/*z* 318 [M–H_2_O] corresponds to the myricetin molecule. Thus, they were tentatively identified as two myricetin derivative isomers and we tentatively identified them as 3,5,7,3′,4′, and 5′-hexahydroxyflavonol. Furthermore, the full mass spectrum of compound **24** (Rt = 14.67 min.) identified in propolis extract, exhibited an intense [M–H]^−^ ion at *m*/*z* 301.26. Compound **24** was assigned as quercetin by comparison of its mass spectrometric data with the literature [26]. Peaks **22** and **23** eluting at 14.01 and 14.29 respectively displayed the same [M–H]^−^ ions at *m*/*z* 315.24. Compounds **22** and **23** were assigned as Isorhamnetin (Quercetin 3′-methyl ether) and Rhamnetin (Quercetin 7-methyl ether) respectively. Due to the similar fragmentation pattern, it is safe to suggest that these two compounds are isomers with the loss of methyl group [27,28].

#### 2.1.5. Flavanols

Compound **15** (Rt = 11.23 min.) exhibited an intense base peak [M–H]^−^ ion at *m*/*z* 319.27 pointed to the molecular formula C_16_H_16_O_7_ and it was tentatively proposed as 4′-O-Methyl-epigallocatechin. The fragment ion *m*/*z* 304 [M–H–CH_3_], the *m*/*z* 301 peak [*m*/*z* 319—H_2_O], and *m*/*z* 137 is the characteristic fragment ion of catechin formed via retro Diels–Alder mechanism [29].

#### 2.1.6. Terpenoids

Compound **27** (C_32_H_46_O_9_), with [M–H]^−^ at *m*/*z* 573.46, showed product ion at *m*/*z* 555 [M–H–H_2_O]^-^, therefore it was tentatively identified as Ganoderic acid Me or ganoderic acid R (triterpenoid). Compounds **26** and **28** were recorded in the mass spectrum (*m*/*z* 583.24) and the fragment ions *m*/*z* 453 at 16.54 and 19.19 min. Based on these results, we suggest that compounds (C_36_H_56_O_6_) are isomers. Thus, they tentatively identified as Oleanolic acid 3-O-(3′-methyl) glutarate or Oleanolic or acid 3-O-(3′,3′-dimethylsuccinate.

### 2.2. In Vitro Antimicrobial Activity

#### 2.2.1. Agar-Based Disk Diffusion Assay

**Figure 1:**
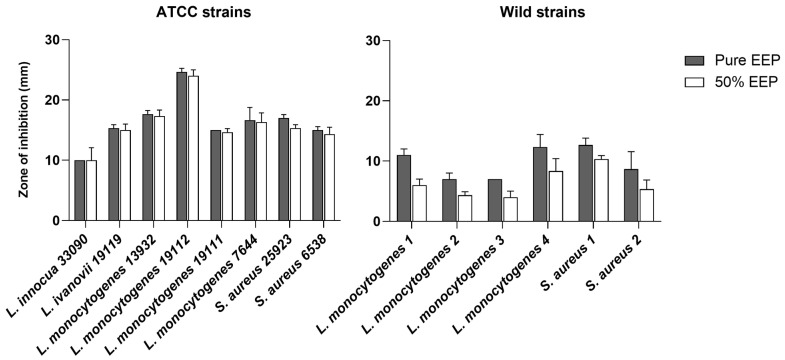
Results of the agar disc diffusion assay for the antimicrobial activity of different concentrations of ethanolic extract of propolis (EEP) from Tunisia. The diameters of the inhibition zone are shown as means and standard deviations of three replicates excluding the diameter of the paper disc (6 mm).

The results of the agar disk diffusion assay, detailed in Figure 1, showed that only Gram-positive bacteria were sensitive to the activity of the EEP while no antibacterial effect was detected against Gram-negative bacteria. These results were observed for both pure and 50% EEP against both wild and ATCC strains. Overall, the diameters of the inhibition zone were significantly wider for the pure EEP than for 50% EEP both against ATCC (*p* = 0.0184) and wild (*p* = 0.0004) strains. As regards the ATCC strains, the antimicrobial activity of both pure and 50% EEP was weak against one *Listeria innocua* strain, moderate against three strains of *L. monocytogenes*, two strains of *Staphylococcus aureus* and one strain of *Listeria ivanovii* while it was high against one strain of *L. monocytogenes*. As regards the wild strains, moderate antimicrobial activity was observed only for pure EEP against one strain of *L. monocytogenes* and one strain of *S. aureus* while against all other strains, the activity was weak both for pure and 50% EEP.

#### 2.2.2. MIC, MBC and MDK_99_

The results obtained for the MIC and the MBC are detailed in Table 1. The MICs obtained for the ATCC strains ranged from 0.0625 to 2.5 mg/mL while it was 0.125 mg/mL against all wild strains tested. As for the MBC, the values were always greater than those obtained for the MIC and ranged from 1.25 mg/mL to 6.25 mg/mL for the ATCC strains while it was 1.25 mg/mL for a wild strain of *L. monocytogenes* and 2.5 mg/mL for all the other wild strains tested.

**Figure 2:**
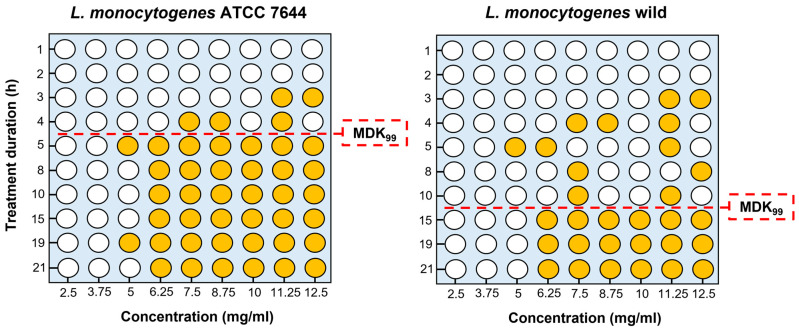
Results of the tolerance of two *L. monocytogenes* strains against an ethanolic extract of propolis (EEP) from Tunisia. Tolerance was evaluated through the determination of the minimum duration for killing the 99% (MDK_99_) of the bacterial populations exposed to the EEP. In yellow, the wells in which bacterial growth was detected and in white, those in which no growth was observed.

Results obtained for the MDK_99_ were graphically reported in Figure 2. In detail, the MDK_99_ was lower for the ATCC strain than for the wild strain and ranged between 4 and 5 h and between 10 and 15 h, respectively. For both strains of *L. monocytogenes*, the antimicrobial activity of EEP appeared to reach saturation at the concentration of 50× MIC.

#### 2.2.3. Antibiofilm Activity

**Figure 3:**
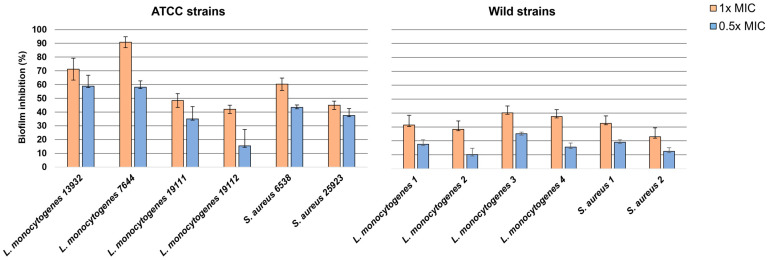
Percentage of biofilm formation inhibition of different concentrations of an ethanolic extract of propolis from Tunisia against several ATCC and wild Gram-positive bacteria.

Results showed that EEP was able to inhibit the formation of biofilm by both ATCC and wild strains at both 1× and 0.5× MIC (Figure 3). The observed differences between the CTL samples and the treated samples were always significant (*p* < 0.05) at both concentrations of EEP. Overall, the antibiofilm activity observed was significantly greater at 1× MIC compared to 0.5× MIC (*p* = 0.0368) in both ATCC and wild strains. The antibiofilm activity was significantly greater against ATCC strains than wild strains (*p* = 0.0067). In detail, the percentage of inhibition of the ATCC strains treated with 1× MIC ranged between 90.82% and 42.03% while for those treated with 0.5× MIC, it was between 58.78% and 15.37%. As regards wild strains, the percentage of inhibition ranged between 22.98% and 40.1% at 1x MIC while it was between 10.15% and 25.14%.

**Figure 4:**
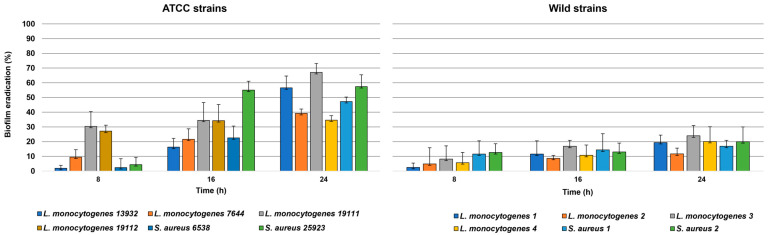
Percentage of biofilm eradication over time of an ethanolic extract of propolis from Tunisia against established biofilm of several ATCC and wild Gram-positive bacteria. The ethanolic extract of propolis was used at a concentration of 1× MIC (see Table 1 for the MIC values of each bacterium).

The EEP at 1× MIC also showed eradication activity against established biofilm against all ATCC and wild strains tested (Figure 4). The eradication rate increased over time against all strains although this trend was not strictly time-dependent. The eradication rate of the preformed biofilm was never 100% after 24 h of treatment in any of the tested bacteria and ranged between a maximum of 67.09% and a minimum of 34.63% for the ATCC strains and between a maximum of 23.98% and a minimum of 11.65% for the wild strains. The antibiofilm activity of EEP was on average significantly higher against ATCC strains than against wild strains after 16 h (*p* = 0.0078) and 24 h (*p* < 0.0001) of treatment while no significant differences were found after 8 h (*p* = 0.7471).

### 2.3. The Use of Ethanolic Extract of Propolis in Salmon Tartare

#### 2.3.1. Storage Test and Sensory Evaluation

The storage test was conducted up to the moment of sensory rejection of the control sample which corresponded to the 10th day (see below).

**Figure 5:**
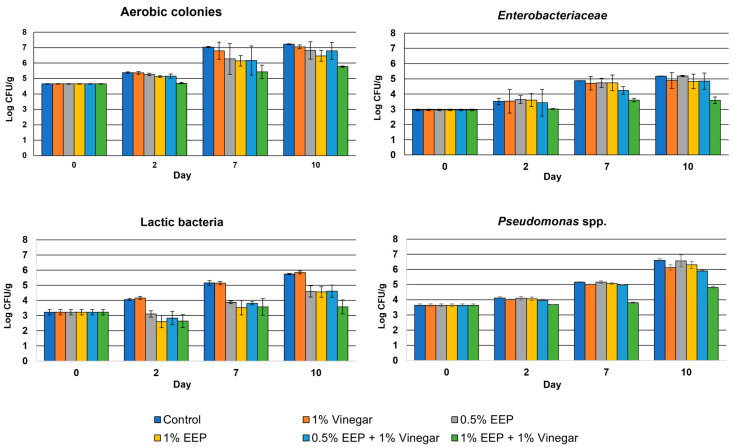
Loads over time of different microbial parameters analyzed in salmon tartare experimentally treated with different formulations of an ethanolic extract of propolis from Tunisia and vinegar.

The results of the microbiological analyses obtained during the storage test are shown in Figure 5.

Overall, the EEP showed significant antimicrobial activity against all the microbiological parameters tested when used at a concentration of 1% and in combination with 1% vinegar. In particular, in the samples treated with 1% EEP + 1% vinegar, an average reduction of 2.18 Log CFU/g for lactic bacteria (*p* = 0.0429), 1.78 Log CFU/g for *Pseudomonas* spp. (*p* = 0.0005), 1.59 Log CFU/g for *Enterobacteriaceae* (*p* = 0.0215) and 1.45 Log CFU/g for aerobic colonies (*p* = 0.0014) were detected compared to loads of the control samples after 10 days of storage. No significant antimicrobial activity was instead observed in the samples treated with only 1% vinegar, in which the loads of all microbiological parameters at no time point differed significantly from those detected in the control samples (*p* > 0.05).

An antimicrobial activity was also observed for the samples treated with 0.5% EEP, 1% EEP and 0.5% EEP + 1% vinegar even if the efficacy was not always significant compared to the loads detected in the control samples.

As regards the samples treated with only 0.5% EEP and 1% EEP without the addition of vinegar, after 10 days of storage, significant antimicrobial activity was observed only against lactic bacteria, which were on average 1.16 Log CFU/g (*p* = 0.0221) and 1.19 Log CFU/g (*p* = 0.0151) lower than the loads detected in the control samples, respectively, while no significant antimicrobial efficacy against aerobic colonies, *Pseudomonas* spp. and *Enterobacteriaceae* was observed.

**Figure 6:**
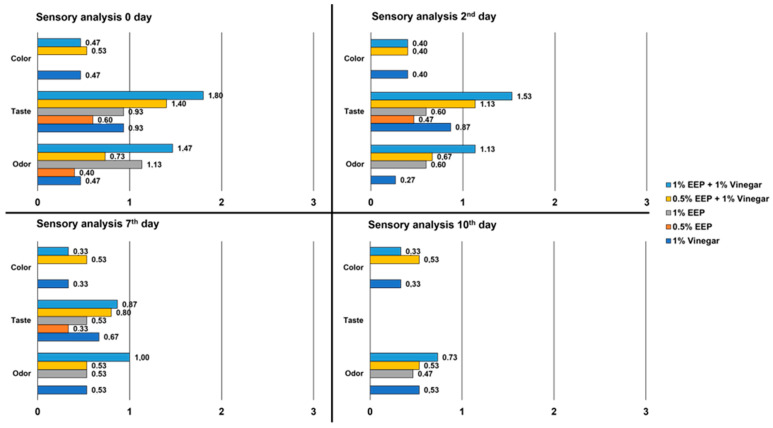
Results of the sensory analysis of salmon tartare experimentally treated with different formulations of an ethanolic extract of propolis from Tunisia and vinegar.

As regards sensory analysis, results are reported in Figure 6. Overall, panellists reported a reduction over time in sensory influence on taste and odor for all treatments tested. The treatments’ influence on color was rated similar at all time points: no influence was ever reported in samples treated only with 1% EEP and 0.5% EEP while for all the other treatments the mean score was always between 0 and 1. Regarding taste, at time 0 and after 2 days, a mean score between 1 and 2 was reported for both treatments at 1% and 0.5% EEP in combination with 1% vinegar. At all-time points, the highest score was attributed to the 1% EEP + 1% vinegar treatment while for the treatments with only EEP or vinegar, the mean taste score ranged from 0 to 1. Regarding odor, at time 0 and after 2 days, a mean score between 1 and 2 was attributed to treatments with 1% EEP alone or in combination with 1% vinegar while the mean score of all the other treatments ranged between 0 and 1 in all time points. The taste results on the 10th day are not reported because the taste of the control sample was such as to compromise its consumption and therefore, we decided to stop the sensory analysis.

#### 2.3.2. Challenge Test to Monitor *Listeria monocytogenes* Growth

**Figure 7:**
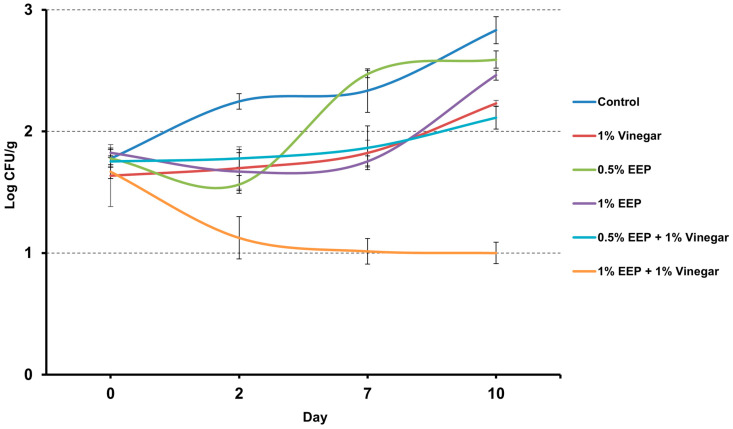
Results of the challenge test performed on salmon tartare experimentally contaminated with *Listeria monocytogenes* and treated with different formulations of an ethanolic extract of propolis from Tunisia and vinegar.

The results obtained from the challenge test are shown in Figure 7. Overall, after 10 days of storage, *L. monocytogenes* grew in all samples except for those treated with 1% EEP + 1% vinegar where a decrease of 0.67 Log CFU/g was detected. The greatest increase was observed in the control sample in which a 1 Log CFU/g increase was observed. After 10 days, no significant antimicrobial activity against *L. monocytogenes* was observed in samples treated with 0.5% EEP (*p* = 0.1931) and 1% EEP (*p* = 0.0755) whose concentrations did not differ significantly from the control sample. On the 10th day, a significant decrease in the growth rate of *L. monocytogenes* was observed also both in samples treated with 1% vinegar alone (*p* = 0.0211) and used in combination with EEP (*p* = 0.0002). Treatment with 1% EEP + 1% vinegar was significantly more effective than treatment with 1% vinegar alone (*p* = 0.0020). In detail, the final concentration of *L. monocytogenes* in the samples treated with 1% vinegar and 0.5% EEP + 1% vinegar was, respectively, 0.65 Log CFU/g and 0.86 Log CFU/g lower than the control sample. The most effective treatment was the 1% EEP + 1% vinegar where a reduction in the *L. monocytogenes* concentration of 1.78 Log CFU/g was detected compared to the control samples.

## 3. Discussion

### 3.1. In Vitro Antimicrobial Activity and Chemical Composition

The biological activities of propolis have long been investigated and there is numerous evidence of its antimicrobial effects. Generally, the antimicrobial activity of EEP was observed to be higher against Gram-positive bacteria than Gram-negative as confirmed by the in vitro analyses of the present study [30]. Indeed, EEP showed antimicrobial efficacy only against Gram-positive bacteria while no activity against Gram-negative was observed (see Figure 1). This was generally attributed to the different structure and composition of the cell wall [31,32]. According to Vadillo-Rodriguez et al. [33], the occurrence of amphiphilic and highly charged molecules in the outer membrane of Gram-negative bacteria, primarily composed of lipopolysaccharides, is likely to inhibit and/or retard the penetration of the antimicrobial components of EEP. As result, higher concentrations of EEP are required to reach the cell membrane and induce cell death. However, comparing our results with those of other studies, the antimicrobial effectiveness against a strain appears highly variable. On one hand, according to our results, several authors report an in vitro antimicrobial efficacy of EEP against different strains of *L. monocytogenes*, *L. innocua* and *S. aureus* with MIC values that varies greatly between studies. On the other hand, in contrast with our results, there is evidence of high in vitro antimicrobial activity against different species of Gram-negative bacteria, including foodborne pathogens such as *Salmonella* Typhimuriumn [34] and *Yersinia enterocolitica* [35]. Antimicrobial activity of EEP was also reported against *E. coli* [36] and *P. aeruginosa* [37] for which no effects were observed in the present study despite the higher concentrations of propolis used. The reason behind these conflicting results could lie in the mechanism of propolis antibacterial activity which is still not fully understood. On one hand, the poor efficacy against Gram-negative bacteria suggests a structural killing mechanism of propolis that induces the release of specific intracellular substances capable of damaging the cellular membrane leading to irreversible cell lysis [33]. On the other hand, some authors reported that the antimicrobial efficacy of propolis would be due to the biological activity of the constituent compounds, such as flavonoids, which perturb protein synthesis preventing bacterial growth and division, thus acting as functional rather than structural disruptors [33].

In this context, it is clear that the chemical composition plays a key role in the antimicrobial efficacy of EEP and would explain why the efficacy against a bacterial strain can vary greatly between propolis with the same concentration but of different origins [38]. In fact, the chemical composition of propolis can vary greatly depending on the region and period of the collection as well as based on its botanical origin [38]. Overall, the chemical profile of the EEP herein tested displayed the typical pattern of an ethanolic extract of propolis (see Table S1) such as pinocembrin, pinobanksin, *p*-Coumaric acids, chrysin, esters of caffeic and ferulic acids [39]. In this regard, these compounds have been identified in various EEP of different geographical origins, such as from Brazil, China, Italy and Spain [40,41,42], and would be responsible for their biological properties. In particular, the antimicrobial efficacy of propolis has been related to the activity of specific constituents that was detected also in the EEP herein tested such as pinobanksin, pinocembrin and *p*-Coumaric acid which are effective against several bacteria [43,44]. Relevant antimicrobial activity against different foodborne pathogens and spoilage microorganisms was also observed for chrysin, ferulic and caffeic acids which were the most predominant compounds in five Polish extract propolis [45]. However, reports on the greater antimicrobial activity of crude propolis compared to blends of its major individual constituents suggests that trace components in the crude propolis are critical for their activity and may have a synergistic effect [43]. In this regard, several studies report how quercetin, which was detected in our EEP, in combination with other flavonoids, shows significant antimicrobial effects against different foodborne pathogens and their ability to form biofilm [30,46,47,48].

In the present study, EEP showed antibiofilm activity against ATCC and wild strains of *L. monocytogenes* and *S. aureus* inhibiting biofilm formation and eradicating the established one (see Figure 3 and Figure 4). Laranjo et al. [49] report an in vitro antibiofilm activity on polyethylene flat-bottom microtiter plates of seven different EEP from Brazil and Portugal against staphylococci isolated from the milk of small ruminants with mastitis, observing a greater efficacy in inhibiting biofilm formation than in its eradication. Dogan et al. [50] report the biofilm inhibition activity of two different EEP from Turkey against ATCC strains of *L. monocytogenes* and *S. aureus*. In detail, the maximum antibiofilm activity percentage of 85% was reported for *L. monocytogenes* ATCC 7644 similar to the results of the present study where the same strain was the most sensible with a percentage of biofilm formation inhibition of 90%. In the present study, the antibiofilm activity of propolis was higher against ATCC strains than against wild strains both for inhibition and eradication activity. To the best of the authors’ knowledge, there are no comprehensive studies that compare the antibiofilm efficacy of EEP between wild-type and certified strains of the same bacterial species. In this regard, several studies evidenced how certain bacterial strains that experienced stress, such as stringent environmental conditions, can develop inheritable or transient defence systems that increase their resistance or tolerance and so their ability to survive bactericidal stress such as exposure to EEP [51]. In this regard, the results obtained for the determination of the MDK_99_ have highlighted how the greater antibiofilm efficacy of EEP against the ATCC strain of *L. monocytogenes* can be related to a greater tolerance of the wild *L. monocytogenes* strain (see Figure 2). This consideration was also confirmed by the fact that both the ATCC and wild-type strain of *L. monocytogenes* had the same MIC. We remember that tolerance can be intended as the general ability of a bacterial population to survive a transient bactericidal treatment [52]. Since all the bacteria are expected to survive transient exposure to bacteriostatic drugs, tolerances can be determined only if bacteria are exposed to bactericidal treatment [52]. This is the reason why we used EEP concentrations that far exceed the MIC and MBC for the determination of the MDK_99_ [53]_._ Results obtained for the MDK_99_ confirmed the higher tolerance of the wild-type strain of *L. monocytogenes* that was killed at least 5 h later than the ATCC when exposed to EEP. These considerations are useful for understanding the real antimicrobial efficacy of propolis and should be taken into consideration to evaluate exposure times to standardize its use in food.

### 3.2. The Use of Ethanolic Extract of Propolis in Salmon Tartare

The antimicrobial properties of propolis and its safety support its application as a biopreservative in the food industry for a wide variety of purposes [54]. Indeed, there is numerous evidence of the great effectiveness of propolis in extending the shelf-life of foods or against foodborne pathogens [54]. EEP was tested in different foods such as meat and poultry products, eggs, milk and dairy products, fruit, vegetables and fruit juices. The use of EEP was also proposed in different fresh fishes and seafood to extend their shelf-life. Payandan et al. [55] tested the effects of different concentrations (3%, 5% and 7%) of ethanolic extracts of Iranian propolis on the microbiological and sensory parameters of minced carp (*Cyprinus carpio*) meat that was stored at 4 °C for 9 days. The obtained results revealed that EEP was efficient against spoilage microorganisms, including aerobic colonies, psychrotrophic populations, lactic acid bacteria, and even *S. aureus*. Propolis was also used in Nile tilapia (*Oreochromis niloticus*) fillets where a decrease in aerobic colonies and psychrophilic bacteria was observed during frozen storage for 6 months [56]. In the present study, the use of 1% or 0.5% EEP in salmon tartare kept under vacuum at refrigeration temperature showed antimicrobial activity only against lactic bacteria while no effects were observed against *Pseudomonas* spp, aerobic colonies and *Enterobacteriaceae* (see Figure 5). In addition, Yazgan et al. [57], as herein observed, reported a significant antimicrobial activity of different EEP concentrations (0.4% and 0.8%) against lactic acid bacteria in vacuum-packed and refrigerated sardine fillets but, contrary to our results, EEP were effective also against psychrotrophic bacteria, coliforms and aerobic colonies. In the present study, significant antimicrobial activity against all the tested parameters was observed only when the EEP was used at 1% and in combination with 1% vinegar (see Figure 5). We could speculate about a synergistic effect between EEP and vinegar resulting in a wider spectrum of antimicrobial activity. The 1% EEP + 1% vinegar was also effective against *L. monocytogenes* which showed a bactericidal activity resulting in a reduction of its concentration over time rather than slowing down its growth rate as instead observed when EEP was used alone (see Figure 7). Other studies have already reported the antilisterial activity of EEP in foods such as in milk where *L. monocytogenes* growth was completely arrested throughout 30 days of storage at 4 °C [58] or in fermented meat sausages where the counts of *L. innocua* decreased 3 Log CFU/g by day 5 [59].

Interestingly, the non-significant reduction in the growth rate of *L. monocytogenes* in samples treated with EEP alone after 10 days of storage might appear in contrast with the results obtained in the in vitro analyses where 1% and 0.5% EEP showed a relevant efficacy against all the tested *Listeria* strains. These conflicting results may be due to the complexity of the food matrix where numerous factors, such as pH, storage conditions or the interaction with other chemical compounds, can affect the activity of EEP and its constituents [32].

Another limitation of using propolis and its extracts in food is represented by the very intense taste and odor which can significantly impact the organoleptic characteristics of the product [45]. Therefore, the addition of propolis in foods is possible only if the concentration necessary to exploit its biological properties does not negatively influence the typical taste and aroma. On one hand, there is evidence that the addition of 0.5% propolis extract to different foods such as fish, sausages, poultry meat products, apple juice, milk and honey finds sensory acceptance [45]. However, in the present study, no significant antimicrobial activity was observed in the salmon tartare treated with 0.5% EEP (see Figure 6). On the other hand, consumer preference relative to EEP at concentrations higher than 0.5% in food varies between products [45]. The addition of 5% propolis in fresh sea bass (*Dicentrarchus labrax*) burgers influenced the odor markedly and its use was only possible after microencapsulation [60]. Instead, Payadan et al. [55] reported a slight improvement in the sensory properties in the case of minced carp meat added with 3%, 5% and 7% of EEP. In the present study, 1% and 0.5% propolis had no more than a barely perceptible influence on the taste and smell of the salmon tartare. When both concentrations were used in combination with vinegar, the influence on taste and aroma was moderate during the first two days of storage. Instead, no color changes were observed in the salmon tartare treated with the different formulation of EEP, probably due to the chromatic affinity with the product. Similar results were obtained by Reis et al. [61] that reported no influence on the color, appearance and texture of burger meat, despite the odor and flavor becoming undesirable. It is noteworthy that in the present study, the influence of the organoleptic characteristics of all the treatments decreased during storage. This could be due to the evaporation of the extract during storage or following the appearance of more marked odors and tastes associated with the product decay which masked the EEP perception.

## 4. Materials and Methods

### 4.1. Sampling of Propolis and Extract Preparation

The raw propolis was collected in March 2021 by scraping the surfaces of *Apis mellifera* beehives located in the city of Tebourba, Tunisia (36°49′46″ N–9°50′28″ E). Once sampled, propolis was stored in sterile opaque plastic containers under refrigeration until the time of the extract preparation. An ethanolic extract of propolis (EEP) was prepared according to a modified protocol of Mello and Hubinger [62]. Crude propolis was grounded in a bench blender and mixed with a solution of ethanol (Sigma Aldrich, St. Louis, MO, USA) and sterile ultrapure water (Sigma Aldrich, St. Louis, MO, USA) (80:20 *v*/*v*). The ethanolic extract was prepared in a ratio of 20% propolis and 80% solvent (*w*/*w*). The obtained mixture was sonicated using an ultrasonic bath at 40 °C for 30 min. and then stored at room temperature in a sterile opaque plastic container for one week, stirring the container manually once a day. Thereafter, the mixture was centrifuged at 8800 rpm for 20 min. and the supernatant was filtered through Whatman No 1 filter paper (Sigma Aldrich, St. Louis, MO, USA), kept under refrigeration for 3 h, and filtered once again for wax removal. The ethanolic extract of propolis thus obtained was stored in the dark in a Duran laboratory bottle (Sigma-Aldrich, St. Louis, MO, USA) at room temperature until the time of analysis.

### 4.2. Chemical Analysis: Qualitative Analysis by UHPLC-ESI/HRMS

The identification of the principal components was performed by using a system of liquid chromatography coupled with electrospray ionization (ESI) and high-resolution mass spectrometry (UHPLC-ESI/HRMS), a Waters ACQUITY UPLC system coupled with a Waters Xevo G2-XS Qtof Mass Spectrometer (Waters Corp., Milford, MA, USA), operating in negative ionization mode. To separate the analytes were used a biphenyl 100 mm × 2.1 mm, 2.6 μm column (Phenomenex, Torrance, CA, USA), and the mobile phase consisting of 0.1% formic acid in water (*v*/*v*) as solvent A and 0.1% formic acid in acetonitrile (*v*/*v*) as solvent B, a flow rate of 0.4 mL min^−1^, and a linear gradient held at 0–1.0 min, 5% B; 1.0–17.0 min., 5–95% B; 17.0–20.0 min., 95%, after each run of 4 min. of wash (95% B), and 5 min. of equilibration was performed before the next sample injection. The autosampler was set to inject 5 μL of sample diluted by a ratio of 1:100. The column was maintained at 30 °C and the UV was set in a range of 210–400 nm. For the ESI source, the following experimental conditions were adopted: electrospray capillary voltage 2.0kV, source temperature 150 °C and desolvation temperature 500 °C. MS spectra were gained by full range in a mass range from 50 to 1200 *m*/*z*. In order to allow HRMS/MS analysis, data-dependent scan (DDA) experiments were performed by selecting the first and the second most intense ions from the HRMS scan event and submitting them to collision-induced dissociation (CID) by applying the following conditions: a minimum signal threshold at 250, an isolation width at 2.0, and normalized collision energy at 30%. Both in full and in MS/MS scan mode, resolving power of 30,000 was used. Before qualitative analysis, the mass spectrometer was calibrated with 0.5 M sodium formate, and leucine-enkephalin (100 pg/μL) was used as LockMass (*m*/*z* 554.2615, 2 kV ionization voltage), infusing concurrently with the flow of column at 10 μL/min and acquired for 1 s each 10 s. The MassLynx software (version 4.2) was used for instrument control, data acquisition, and data processing.

### 4.3. In Vitro Antimicrobial Activity

#### 4.3.1. Preparation of the Strains

The antimicrobial activity of the EEP was tested against different ATCC and wild bacterial strains reported in Table 2. The wild strains have been previously identified by Matrix-Assisted Laser Desorption/Ionization-Time of Flight (MALDI-TOF) Mass Spectrometry (MS). A Vitek Mass Spectrometer Axima Assurance (bioMérieux, Firenze, Italy) was used with the following settings: positive linear mode, laser frequency of 50 Hz, acceleration voltage of 20 kV and extraction delay time of 200 ns. The mass spectra range was set to detect from 2000 to 20,000 Da. MALDI-TOF MS generated unique mass spectra for each tested colony, which were transferred into the SARAMIS software (Spectral Archive and Microbial Identification System, database version V4.12, software year 2013, bioMérieux, Firenze, Italy) and compared to the database of reference bacteria spectra and super spectra, obtaining identification at the genus and species levels. Only a match of at least 70% was considered reliable. All the strains were stored at –80 °C at the microbial collection of the “Food Microbiology Laboratory” of the Department of Veterinary Sciences, University of Messina (Messina, Italy). The tested strains were prepared by plating a loopful of the frozen stock into Tryptone Soy Agar plates (TSA; Biolife, Milan, Italy) and incubated overnight at 37 °C for 24 h before each analysis.

#### 4.3.2. Agar-Based Disk Diffusion Assay

The antimicrobial activity of the EEP was preliminarily tested by the agar diffusion method inspired by Mazzarino et al. [63].

For each strain, several colonies from overnight culture on TSA were picked with a sterile loop and suspended in a solution of peptone water saline (PWS; Biolife, Milan, Italy) to obtain a final turbidity of 0.5 McFarland (~10^8^ CFU/mL). A spectrophotometer (Biosigma, Cona, Italy), previously calibrated against a 0.5 McFarland turbidity standard, was used to adjust the density of the suspensions by adding SP or more bacteria. Suspensions were used immediately after preparation and never beyond 15 min. A total of 500 µL from each suspension was inoculated on plates of Mueller-Hinton agar (MH; Biolife, Milan, Italy) and swabbed in three directions ensuring that there were no gaps between streaks. A 10 μL drop of EEP at different concentrations (pure, 50%) was deposited on cellulose disc filters (6 mm in diameter; Biolife, Milan, Italy) which were placed individually on the MH plates within 15 min. of inoculation. The pure drop of EEP contained 2 mg of propolis while those with 50% EEP contained 1 mg of propolis. Then, the plates were incubated overnight at 37 °C. The diameter of the inhibition zones was measured with a Vernier calliper with a minimum resolution of 0.005 mm and expressed as the mean ± standard deviation of three replicates using 80% ethanol as negative control. The antibacterial activity was classified into three levels based on the diameter of the inhibition zone: weak (inhibition zone ≤ 12.0 mm), intermediate (12.1 mm ≤ inhibition zone ≤ 20.0 mm) and marked (inhibition zone ≥ 20.1 mm).

#### 4.3.3. Minimum Inhibitory Concentration (MIC) and Minimum Bactericidal Concentration (MBC)

The MIC and MBC of EEP were evaluated only for those bacteria which were sensitive to the EEP antimicrobial activity in the previous agar-based disk diffusion assay (regardless of the inhibition zone diameter; see paragraph 4.3.2).

The MIC was evaluated by the broth microdilution method according to Kowalska-Krochmal and Dudek-Wicher [64]. A 96-microwell plate (Biosigma, Cona, Italy) was filled with decreasing concentrations of EEP (25, 12.5, 6.25, 5, 2.5, 1.25, 0.25, 0.625, 0.125, 0.0625, 0.0125 mg/mL) prepared in 100 µL of Tryptic Soy Broth (TSB; Biolife, Milan, Italy). A fresh inoculum of each bacteria was grown in TSB at 37 °C for 24 h. Subsequently, a microbial suspension from the broth cultures was inoculated in the microwell plates obtaining a final concentration of ~10^4^ CFU/mL in each well. Then, the microwell plates were incubated a 37 °C for 24 h. The positive control consisted of broth medium without EEP inoculated with microbial suspensions while the uninoculated broth medium with the EEP served as the negative control. The lowest EEP concentration in which there was no visible growth (turbidity of the broth medium) was considered to be the MIC.

The MBC was determined by inoculating the suspensions from each microwell on TSA plates incubated at 37 °C for 24 h. The lowest concentration in which there was no microbial growth was considered to be the MBC.

The MIC and MBC analyses were performed in triplicates.

#### 4.3.4. Tolerance Evaluation: Minimum Duration for Killing the 99% (MDK_99_) of the Bacterial Population

In the present study, the tolerance of an ATCC strain and a wild strain of *L. monocytogenes* against EEP was determined and compared based on the protocol proposed by Brauner et al. [53].

Strains of *L. monocytogenes* with an equal MIC value (ATCC 7644—*L. monocytogenes* 4) were selected for the present investigation.

A total of 90 Eppendorfs (Biosigma, Cona, Italy; 2 mL volume) were arranged in rows on a rack and filled with decreasing concentrations of EEP (100×, 90×, 80×, 70×, 60×, 50×, 40×, 30×, 20× MIC) prepared in 100 µL of TSB. Eppendorfs without EEP were used as control. The choice to test very high concentrations of EEP is not accidental and it is crucial for the reliability of the test results. Indeed, MDK_99_ is a relevant parameter for assessing tolerance as soon as the antibacterial activity is only slightly dependent on the compound concentration. This typically occurs at high concentrations when the compound efficacy reaches saturation and the antimicrobial activity mostly depends on the duration of the treatment. We do not know exactly at which concentrations the EEP activity will reach saturation and that is why we use decreasing concentrations.

A fresh inoculum of bacteria, grown under shaking on TSB at 37 °C for 24 h, was diluted with fresh broth and inoculated into the Eppendorf one row at a time after 1, 2, 3, 4, 5, 8, 10, 15, 19 and 21 h in order to obtain a final concentration of 100 bacteria (10^2^ CFU/100 µL) in each. The Eeppendorfs were left inside an oscillating incubator (Vdrl Asal 711/CT, Bioltecnical service, Italy) to reach the temperature of 37 °C before the first inoculation. Once all rows had been inoculated, the Eppendorfs were centrifuged all together for 10 min. at 1200× *g* at 10 °C and the supernatant was manually discarded to wash off the EEP. This procedure was repeated twice by adding an equal volume of fresh broth before the second spin. As reported for antibiotics, we assumed that each spin reduced the concentration of EEP by 10–20 times ensuring its removal even at the highest concentration (100× MIC) used in this study. After that, the Eppendorfs were filled with fresh broth and incubated under shaking at 37 °C for 3 days in order to ensure the growth of even those bacterial cells characterized by slower growth. The growth evaluation was performed by inoculating the suspensions from each Eppendorf on TSA plates incubated at 37 °C for 24 h.

The lack of growth In the TSA plates indicates that > 99% of the bacteria present in each Eppendorf died since colony growth implied that at least one out of 100 bacteria had survived the treatment. At the end of the analysis, we will obtain a grid of an equal concentration of bacteria that have been exposed for different times to high concentrations of EEP. Once the EEP was washed off, only those bacteria that survived the treatment will be able to grow first in the fresh broth and then on the plates.

On this background, it is possible to estimate the MDK_99_ that will be included in that time range in which microbial growth is no longer observable.

#### 4.3.5. Antibiofilm Activity

The antibiofilm activity of EEP was tested against 4 ATCC (13932, 7644, 19111, 19112) and 4 wild strains of *L. monocytogenes* and 2 ATCC (6538, 25923) and 2 wild strains of *S. aureus*. The evaluation was carried out in vitro inspired by the protocol developed by Gao et al. [65] as follows.

##### The Biofilm Inhibition Assay

The bacterial strains were grown overnight in TSB at 37 °C and subsequently diluted with fresh broth to obtain a final concentration of ~10^7^ CFU/mL. The broth cultures were inoculated into microtiter plates (Biosigma, Cona, Italy) by filling each well with 4 mL of the bacterial suspension. A sterile square glass coverslip (6 mm side) was embedded in each well and EEP with different concentrations was added to each well (final concentrations of 0.5× MIC and 1× MIC in each well). Coverslips exposed only to the bacterial suspension without EEP were used as control. Then, the microtiter plates were incubated at 37 °C for 24 h. After that, the microbial suspensions were removed from each well and the coverslips were gently washed thrice with phosphate-buffered saline (PBS) to remove planktonic cells. The biofilm attached to the coverslips was stained with crystal violet (Sigma Aldrich, MO, USA) for 30 min. at room temperature, washed with PBS and air-dried. Once stained, the crystal violet and biofilm were dissolved by absolute ethanol (Sigma Aldrich, MO, USA) and their absorbance (OD) was measured with a spectrophotometer (Biosigma, Cona, Italy) set at 630 nm. The analyses were performed in triplicate. The percentage of biofilm inhibition was calculated according to the following equation

Biofilm inhibition (%) = [(OD_control_ − OD_sample_)/OD_control_] × 100 (1)

##### The Biofilm Eradication Assay

Bacterial cultures grown overnight in TSB were diluted 100-fold using fresh broth into microtiter plates (Biosigma, Cona, Italy) filling each well with 4 mL of the bacterial suspension. Sterile square glass coverslips were embedded in each well and the microtiter plates were incubated at 37 °C for 24 h. Subsequently, the bacterial suspension was gently removed and the fresh broth was added to each well. This procedure was repeated 3 times, once a day for three consecutive days in order to make the biofilm mature. Thereafter, the bacterial suspension was removed and each well was filled with 4 mL of fresh broth containing EEP (final concentration of 1× MIC in each well). In this test, only a single concentration of the EEP (1× MIC) was used differently than in “The biofilm inhibition assay” (see above; 0.5× MIC and 1× MIC) since preliminary tests showed a poor biofilm eradication efficacy at 0.5× MIC (data not show). Therefore, considering the time and effort required to carry out the assay, it was preferred to perform the test considering only a single concentration. Wells filled only with fresh broth without EEP were used as positive CTLs that were processed as the other samples. Then, the microtiter plates were incubated at 37 °C and, after 8 h, 16 h and 24 h, the coverslips were processed as already described above (see above “The biofilm inhibition assay”). The analyses were performed in triplicate. The percentage of biofilm eradication was calculated over time according to the following Equation (2):

Biofilm eradication (%) = [(OD_control_ − OD_sample_)/OD_control_] × 100 (2)

### 4.4. In Situ Analysis: Evaluation of the Use of Ethanolic Extract of Propolis in Salmon Tartare

#### 4.4.1. Preparation of Salmon Tartare Samples

Fresh salmon tartare was experimentally added with different concentrations of EEP to evaluate any antimicrobial activity that would justify its possible use in food. Salmon tartare was chosen for this survey as the addition of natural additives to fresh salmon, such as herbs and their extracts, is a common practice in some famous culinary preparations such as gravlax: a traditional Swedish dish consumed worldwide in which fresh salmon is marinated with salt, sugar and dill and for which numerous variations were proposed based on the ingredients added to the marinade.

About 2500 g of fresh salmon tartare was prepared from defrosted salmon fillets cut into small cubes with the aid of sterile knives. Six aliquots of equal weight were prepared: one aliquot represented the negative control without EEP while the remaining 5 aliquots were added one with 1% vinegar and 1% EEP, one with 1% vinegar and 0.5% EEP, one with 1% vinegar alone, one with 1% EEP alone and one with 0.5% EEP alone. Vinegar and EEP were added directly to the salmon cubes which were then mixed with the help of sterile wooden sticks. Once prepared, each aliquot was divided into two sub-aliquots destined for two distinct analyses: a storage test and a challenge test which are described below.

#### 4.4.2. Storage Test and Sensory Evaluation

The present investigation aimed to establish the shelf-life of the variously treated salmon tartare by evaluating whether the antimicrobial effects of EEP and its sensory influences allow the product to be preserved longer without compromising its organoleptic quality. Once prepared, each of the 6 salmon tartare sub-aliquots was divided into three batches which were individually vacuum-packed and then stored under refrigeration. The three batches (called “samples” hereinafter) were prepared in order to perform the analyzes in triplicate at each time point.

Samples were then processed periodically after 0, 2, 7 and 10 days of storage for the evaluation of the following microbiological parameters: (*i*) enumeration of the aerobic colonies at 30 °C (ISO 4833-1:2013) on plates of Plate Count Agar (Biolife, Milano, Italy) incubated at 30 °C ± 1 °C for 72 h [66]; (*ii*) enumeration of the *Enterobacteriaceae* (ISO 21520:2017) on plates of Violet Red Bile Glucose Agar (Biolife, Milan, Italy) incubated at 37 ± 1 °C for 24 h [67]; (*iii*) detection and enumeration of *Pseudomonas* spp. (ISO 13720:2010) on plates of Pseudomonas Agar Base (HiMedia Laboratories, Mumbai, India; added with CFC *Pseudomonas* Supplement) incubated at 25 ± 1 °C for 48 h [68]; (*iv*) enumeration of mesophilic lactic acid bacteria (ISO 15214:1998) on plates of M.R.S. Agar (Biolife, Milan, Italy) incubated at 30 ± 1 °C for 72 h [69].

The samples were processed by diluting an amount of ~10 g with buffered peptone water (Biolife, Milano, Italy) in a ratio of 1:9 *w*/*v*. Once homogenized by a stomacher (400 Circulator; International PBI s.p.a., Milano, Italy) for 60 s at 230 rpm, samples were then processed for the evaluation of the microbiological parameters stated above.

A portion of each sample was used to perform a sensory analysis. Five people (two women and three men) selected among the staff of the Laboratory of Inspection of Food of Animal Origin, Department of Veterinary Sciences, University of Messina (Messina, Italy) were involved in the sensory evaluation of the samples that was performed according to ISO 6658:2017 and ISO 8589:2007 [70,71]. All members had previously trained in the sensory evaluation of fresh salmon tartare according to ISO 5492:2008 and ISO 8586:2012 [72,73]. In particular, six 1-h sessions were used to familiarize the panellists with the sensory characteristics of the salmon tartare which were shown at different stages of freshness. An amount of ~5 g of each salmon tartare sample was placed on white plates for 5 min at room temperature and then offered to the panellist under a white light regime. The panellists were asked to express an evaluation of the ‘color’, ‘odor’ and ‘taste’ of the treated samples by comparing them with the control sample and evaluating the sensory influence of treatments by assigning to each descriptor a score that could be 0 if ‘typical’, 1 if ‘just perceptible’, 2 if ‘moderate’ and 3 if ‘intense’. It is important to underline that the sensory evaluation herein performed was based on a hedonistic analysis that requires a much greater number of participants than those involved in this study. Therefore, the present assessment was performed for exploratory purposes only as more extensive and in-depth analyses are needed to understand how the tested EEP affects the sensory profile of the salmon tartare.

#### 4.4.3. Challenge Test

The other 6 salmon tartare sub-aliquots were experimentally contaminated with *L. monocytogenes* to evaluate the antimicrobial efficacy of EEP against this foodborne pathogen which represents one of the main microbiological safety hazards of salmon tartare. A strain of *L. monocytogenes* ATCC 7644 was resumed by inoculating a cryobed from a frozen stock into 10 mL of TSB incubated at 37 °C overnight. Growth of the broth culture was followed through a spectrophotometer and, once a concentration of ~10^7^ CFU/mL was reached, ten-fold dilutions were performed in fresh broth to obtain a final concentration of ~10^3^ CFU/mL. The resulting broth culture was incubated under refrigeration up to reaching a concentration of ~10^7^ CFU/mL and then ten-fold diluted in peptone saline water down to a concentration of ~10^4^ CFU/mL. The broth culture thus obtained was used to experimentally contaminate the 6 salmon tartare sub-aliquots using an inoculum of 1 mL per 50 g of salmon tartare. Once contaminated, each of the 6 salmon tartare sub-aliquots was divided into three batches which were individually vacuum-packed and then stored under refrigeration. The three batches (called “samples” hereinafter) were prepared in order to perform the analyzes in triplicate at each time point. Samples were periodically processed after 0, 2, 7 and 10 days of storage as described above (see Section 4.4.2) for the enumeration of *L. monocytogenes* (ISO 11290-2:2017) on Agar Listeria according to Ottaviani & Agosti (Biolife, Milano, Italy) and Listeria Palcam Agar (Biolife, Milano, Italy) both incubated at 37 ± 1 °C for 24–48 h [74].

### 4.5. Statistical Analysis

The statistical package Graph Pad Prism 9 (San Diego, CA, USA) for Windows was used for data processing.

The T-test was performed to compare the antibacterial efficacy of the different EEP concentrations used in the agar-based disk diffusion assay and the biofilm inhibition assay. The Two-way analysis of variance (ANOVA) was used to compare the antibiofilm activity of EEP over time against ATCC and wild strains in the biofilm eradication assay. Statistical analyzes in both antibiofilm activity tests were carried out considering the means of the results obtained with the spectrophotometer. MIC, MBC and MDK_99_ values were treated as ordinal numerical variables. The Two-way ANOVA was also used to analyze data obtained in the storage and challenge tests. In this regard, any significant differences in the antibacterial efficacy of the different EEP formulations (different concentrations with and without vinegar) were evaluated by comparing the results obtained for each microbiological parameter at each time point. Tukey’s honestly significant difference test was used for the multiple comparisons within the obtained ANOVA data. The normal distribution of data for each microbiological parameter was verified by the D’Agostino–Pearson omnibus test. The critical significance level (*p*) was set at 5% (0.05), and all tests were two-sided.

## 5. Conclusions

The findings of the present study have highlighted the antimicrobial properties of an ethanolic extract of propolis from Tunisia. The antimicrobial and antibiofilm efficacy, related to the phenolic constituents, was observed in vitro only against Gram-positive bacteria, both ATCC and wild strains. The antimicrobial activity was also tested in salmon tartare at a concentration of 1% in association with 1% vinegar resulting in a low sensory influence on the product and high antimicrobial efficacy both against spoilage bacteria and *L. monocytogenes*. In this background, propolis could be a suitable natural alternative to common preservatives for ensuring safety and enhancing the quality of raw or practically raw salmon-based products. However, its use in food on an industrial scale would require the availability of large quantities and, therefore, the need to reduce production and management costs. In this perspective, further studies are needed to improve propolis supply availability, increase its antimicrobial efficacy while reducing the sensory influence and understand its stability during storage.

## Figures and Tables

**Table 1:** Minimum inhibitory concentrations (MIC) and minimum bactericidal concentrations (MBC) of ethanolic extract of propolis from Tunisia against different ATCC and wild Gram-positive bacteria. Results are expressed as mg/mL.

Strain		MIC	MBC
ATCC	*L. innocua* 33090	0.625	12.5
	*L. ivanovii* 19119	2.5	12.5
	*L. monocytogenes* 13932	0.25	2.5
	*L. monocytogenes* 19112	0.125	6.25
	*L. monocytogenes* 19111	0.125	1.25
	*L. monocytogenes* 7644	0.125	1.25
	*S. aureus* 25923	0.0625	2.5
	*S. aureus* 6538	0.125	2.5
Wild	*L. monocytogenes* 1	0.125	2.5
	*L. monocytogenes* 2	0.125	2.5
	*L. monocytogenes* 3	0.125	2.5
	*L. monocytogenes* 4	0.125	1.25
	*S. aureus* 1	0.125	2.5
	*S. aureus* 2	0.125	2.5

**Table 2:** ATCC and wild bacterial strains tested against the ethanolic extract of propolis.

Strains		
	ATCC	Wild (Origin)
Gram-positive	*Listeria innocua* 33090	*Listeria monocytogenes* 1 (smoke salmon)
	*Listeria ivanovii* 19119	*Listeria monocytogenes* 2 (smoked tuna)
	*Listeria monocytogenes* 13932	*Listeria monocytogenes* 3 (smoked swordfish)
	*Listeria monocytogenes* 19112	*Listeria monocytogenes* 4 (aged cheese)
	*Listeria monocytogenes* 19111	*Staphylococcus aureus* 1 (aged cheese)
	*Listeria monocytogenes* 7644	*Staphylococcus aureus* 2 (smoked salmon)
	*Staphylococcus aureus* 25923	
	*Staphylococcus aureus* 6538	
Gram-negative	*Salmonella* Enteridis *13076*	*Salmonella* Enteritidis (eggs)
	*Salmonella* Typhimurium *14028*	*Salmonella* Typhimurium (pork meat)
	*Pseudomonas aeruginosa 27853*	*Pseudomonas fluorescens* (fresh cheese)
	*Escherichia coli 35218*	*Escherichia coli* (milk)
	*Escherichia coli 25922*	

## Data Availability

Not applicable.

## Supplementary Materials

The following supporting information can be downloaded at: https://www.mdpi.com/article/10.3390/antibiotics12050802/s1, Table S1 Chemical composition. References [75,76,77,78] are cited in the Supplementary Materials.
