# Supplementary material for: Chemical Composition and Comprehensive Antimicrobial Activity of an Ethanolic Extract of Propolis from Tunisia
Source: Antibiotics (Basel). 2023 Apr 23;12(5):802. doi: 10.3390/antibiotics12050802 (PMC10215150; doi:10.3390/antibiotics12050802)
Supplement: Supplementary file 1 [file antibiotics-12-00802-s001.zip › antibiotics-2345354-supplementary.pdf]

Table S1: Chemical composition. UHPLC-HRMS data of compounds detected in the ethanolic exctracts of propolis from Tunisia.

Peak	Tr (min)	[M-H]-	Molecular formula	Fragmentation pattern	Tentative identification	Reference
1	5.83	623.20	$C_{28}H_{32}O_{16}$	271	Dimethoxy-naringenin diglucoside	Ghallab et al., 2021
2	6.71	609.1854	$C_{28}H_{34}O_{15}$	301	Hesperidin	Taamalli et al.,2015
3	7.35	359.19	$C_{19}H_{20}O_7$	341, 299, 163, 119	*p*-coumaric acid derivative	Saftic et al., 2018, Svečnjak et al., 2020
4	7.89	353.27	$C_{16}H_{18}O_9$	191, 179	Caffeoyl quinic acid	Ghalleb et al., 2021
5	7.98	285.0759	$C_{16}H_{14}O_5$	253, 271	Pinobanksin-methyl-ether	Bertrams et al., 2013
6	8.05	315.0875	$C_{17}H_{16}O_6$	255, 151	Dihydroxy-dimethoxyflavone	Vieira de Morais et al., 2021
7	8.67	271.0610	$C_{15}H_{12}O_5$	253, 151	Pinobanksin	Sartori et al., 2022
8	8.96	299.0548	$C_{16}H_{12}O_6$	255,227	Hispidulin	Vieira de Morais et al., 2021
9	9.14	329.0657	$C_{17}H_{14}O_7$	299, 271, 243	tricin	Vieira de Morais et al., 2021
10	9.34	335.26	$C_{15}H_{12}O_9$	318, 317	3,5,7,3',4',5'-hexahydroxy-flavonol (myricetin derivative) isomer	Viscardi et al., 2017
11	10.35	335.23	$C_{15}H_{12}O_9$	318	3,5,7,3',4',5'-hexahydroxy-flavonol (myricetin derivative) isomer	Viscardi et al., 2017
12	10.41	253.0495	$C_{15}H_{10}O_4$	119	Chrysin	Vieira de Morais et al., 2021
13	10.85	255.0662	$C_{15}H_{12}O_4$	213, 151	Pinocembrin	Bertrams et al., 2013
14	11.06	313.0721	$C_{17}H_{14}O_6$	253, 271	Pinobanksin-acetate	Bertrams et al., 2013
15	11.23	319.27	$C_{16}H_{16}O_7$	304, 301, 137	4'-O-Methyl-epigallocatechin	Meng et al., 2001; Jeong et al., 2003
16	11.37	327.0873	$C_{18}H_{16}O_6$	271, 253	Pinobanksin-propionate	Sartori et al., 2022
17	11.82	355.19	$C_{20}H_{20}O_6$	271, 253, 211	Pinobanksin-3-O-pentanoate	Ghallab et al., 2021
18	12.13	325.19	$C_{15}H_{18}O_8$	187,145, 163	*p*-coumaric acid derivative	Svečnjak et al., 2020
19	12.14	325.19	$C_{15}H_{18}O_8$	187,145, 163	*p*-coumaric acid derivative	Svečnjak et al., 2020
20	12.43	385.20	$C_{20}H_{18}O_8$	341, 282, 193	Ferulic acid dimer	Ahmed et al., 2017;
21	12.95	347.22	$C_{16}H_{12}O_9$	169, 125, 124	Gallic acid derivative	Pierson et al., 2014
22	14.01	315.24	$C_{16}H_{12}O_7$	300, 284, 161	Isorhamnetin (Quercetin 3′-methyl ether)	Castro et al. 2014; Saftíc et al., 2018
23	14.29	315.24	$C_{16}H_{12}O_7$	300, 271	Rhamnetin (Quercetin 7-methyl ether)	Castro et al. 2014
24	14.67	301.26	$C_{15}H_{10}O_7$	179, 151	Quercetin	Yuan et al., 2020
25	15.81	383.39	$C_{21}H_{20}O_7$	163, 145, 119	*p*-coumaric acid derivative	Saftíc et al., 2018; Svečnjak et al., 2020
26	16.54	583.24	$C_{36}H_{56}O_6$	453	Oleanolic acid 3-O-(3'-methyl)glutarate or Oleanolic* acid 3-O-(3',3'-dimethyl)succinate (terpenoid)	-
27	17.12	573.46	$C_{32}H_{46}O_9$	555	Ganoderic acid R or Me(triterpenoid)	Sahlan et al., 2021
28	19.19	583.48	$C_{36}H_{56}O_6$	453	Oleanolic acid 3-O-(3'-methyl)glutarate or Oleanolic* acid 3-O-(3',3'-dimethyl)succinate (terpenoid)	-

References

1. Ghallab, D.S.; Mohyeldin, M.M.; Shawky, E.; Metwally, A.M.; Ibrahim, R.S. Chemical profiling of Egyptian propolis and determination of its xanthine oxidase inhibitory properties using UPLC–MS/MS and chemometrics. *Lwt* **2021**, *136*, 110298.

2. Taamalli, A.; Arráez-Román, D.; Abaza, L.; Iswaldi, I.; Fernández-Gutiérrez, A.; Zarrouk, M.; Segura-Carretero, A. LC-MS-based metabolite profiling of methanolic extracts from the medicinal and aromatic species *Mentha pulegium* and *Origanum majorana*. *Phytochem Anal* **2015**, *26(5)*, 320-330.
3. Saftić, L.; Peršurić, Ž.; Fornal, E.; Pavlešić, T.; Pavelić, S.K. Targeted and untargeted LC-MS polyphenolic profiling and chemometric analysis of propolis from different regions of Croatia. *J Pharm Biomed* **2019**, *165*, 162-172.
4. Svečnjak, L.; Marijanović, Z.; Okińczyc, P.; Marek Kuś, P.; Jerković, I. (). Mediterranean propolis from the adriatic sea islands as a source of natural antioxidants: Comprehensive chemical biodiversity determined by GC-MS, FTIR-ATR, UHPLC-DAD-QqTOF-MS, DPPH and FRAP assay. *Antioxidants* **2020**, *9(4)*, 337.
5. Bertrams, J.; Kunz, N.; Müller, M.; Kammerer, D.; Stintzing, F.C. Phenolic compounds as marker compounds for botanical origin determination of German propolis samples based on TLC and TLC-MS. *J Appl Bot Food Qual* **2013**, *86(1)*, 134-138.
6. Vieira de Morais, D.; Rosalen, P.L.; Ikegaki, M.; de Souza Silva, A.P.; Massarioli, A.P.; de Alencar, S.M. Active antioxidant phenolics from Brazilian red propolis: An optimization study for their recovery and identification by LC-ESI-QTOF-MS/MS. *Antioxidants* **2021**, *10(2)*, 297.
7. Sartori, G.; Pesarico, A.P.; Pinton, S.; Dobrachinski, F.; Roman, S.S.; Pauletto, F.; Rodrigues, L.C.J.; Prigol, M. Protective effect of brown Brazilian propolis against acute vaginal lesions caused by herpes simplex virus type 2 in mice: involvement of antioxidant and anti-inflammatory mechanisms. *Cell Biochem* **2012**, *30(1)*, 1-10.
8. Viscardi, D.Z.; Oliveira, V.S.D.; Arrigo, J.D.S.; Piccinelli, A.C.; Cardoso, C.A.; Maldonade, I.R.; Kassuya, C.A.L.; Sanjinez-Argandoña, E.J. Anti-inflammatory, and antinociceptive effects of Campomanesia adamantium microencapsulated pulp. *Rev Bras de Farmacogn* **2017**, *27*, 220-227.
9. Meng X.; Lee M.J.; Li C.; Sheng S.; Zhu N.; Sang S.; Ho C.T.; Yang, C.S. Formation and Identification of 4′-O-Methyl-(−)-Epigallocatechin in Humans. *Drug Metab Dispos* **2001**, *29(6)*, 789-793.
   - Jeong, C.H., Bae, Y.I., Lee, H.J., Shim, K.H. Chemical components of propolis and its ethanolic extracts. *J Korean Soc Food Sci Nutr* **2003**, *32(4)*, 501-505.
10. Ahmed, E.F.; Elkhateeb, W.A.; Taie, H.A.; Rateb, M.E.; Fayad, W. Biological capacity and chemical composition of secondary metabolites from representatives Japanese lichens. *J App Pharmac Sci* **2017**, *7(1)*, 098-103.
11. Pierson, J.T.; Monteith, G.R.; Roberts-Thomson, S.J.; Dietzgen, R.G.; Gidley M.J.; Shaw P.N. Phytochemical extraction, characterisation and comparative distribution across four mango (*Mangifera indica* L.) fruit varieties. Food Chem **2014**, *149*, 253-263.
12. Castro, C.; Mura, F.; Valenzuela, G.; Figueroa, C.; Salinas, R., Zuñiga, M.C.; Torres, J.L.; Fuguet, E.; Delporte, C. Identification of phenolic compounds by HPLC-ESI-MS/MS and antioxidant activity from Chilean propolis. Food Res Int **2014**, *64*, 873-879.
13. Yuan, M.; Yuan, X.J.; Pineda, M.; Liang, Z.Y.; He, J.; Sun, S.W.; Pan, T.L.; Li, K. P. A comparative study between Chinese propolis and Brazilian green propolis: metabolite profile and bioactivity. *Food Funct* **2020**, *11(3)*, 2368-2379.
14. Sahlan, M.; Hapsari, N.R.A.; Pratami, K.D.; Khayrani A.C.; Lischer, K.; Alhazmi, A.; Mohammedsaleh, Z.M.; Shater, A.F.; Saleh, F.M.; Alsanieh, W.F.; Sayed, S.; Gaber, A. Potential hepatoprotective effects of flavonoids contained in propolis from South Sulawesi against chemotherapy agents. *Saudi J Biol Sci* **2021**, *28*, 5461–5468.
